# The gas cylinder, the motorcycle and the village health team member: a proof-of-concept study for the use of the Microsystems Quality Improvement Approach to strengthen the routine immunization system in Uganda

**DOI:** 10.1186/s13012-015-0215-3

**Published:** 2015-03-08

**Authors:** Dorothy A Bazos, Lea R Ayers LaFave, Gautham Suresh, Kevin C Shannon, Fred Nuwaha, Mark E Splaine

**Affiliations:** 1 Community Engagement, the Prevention Research Center at Dartmouth, The Dartmouth Institute for Health Policy and Clinical Practice, Dartmouth College, 35 Centerra Parkway, Lebanon, NH 03766 USA; 2 JSI Research & Training Institute, Inc., Community Health Institute, 501 South Street, 2nd Floor, Bow, NH 03304 USA; 3 Pediatrics and Community & Family Medicine, Geisel School of Medicine, Dartmouth-Hitchcock Medical Center, 1 Rope Ferry Road, Hanover, NH 03755 USA; 4 SAC Health System, Department of Family Medicine, Loma Linda University School of Medicine, Suite 206-A, Loma, Linda, CA 92354 USA; 5 Disease Control and Prevention, Makerere University School of Public Health, PO Box 7072, Kampala, Uganda; 6 The Dartmouth Institute for Health Policy and Clinical Practice and Community and Family Medicine, Geisel School of Medicine at Dartmouth, 30 Lafayette Street, Lebanon, NH 03766 USA; 7 501 South Street, Bow, NH 03304 USA

**Keywords:** Quality improvement, Uganda, Routine immunization, Vaccination, Action learning collaborative, Microsystem, Systems thinking, Systems strengthening, Innovation

## Abstract

**Background:**

Although global efforts to support routine immunization (RI) system strengthening have resulted in higher immunization rates, the World Health Organization (WHO) estimates that the proportion of children receiving recommended DPT3 vaccines has stagnated at 80% for the past 3 years (WHO Fact sheet—Immunization coverage 2014, WHO, 2014). Meeting the WHO goal of 90% national DPT3 coverage may require locally based strategies to support conventional approaches. The Africa Routine Immunization Systems Essentials-System Innovation (ARISE-SI) initiative is a proof-of-concept study to assess the application of the Microsystems Quality Improvement Approach for generating local solutions to strengthen RI systems and reach those unreached by current efforts in Masaka District, Uganda.

**Methods:**

The ARISE-SI intervention had three components: health unit (HU) advance preparations, an action learning collaborative, and coaching of improvement teams. The intervention was informed and assessed using qualitative and quantitative methods. Data collection focused on changes and outcomes of improvement efforts among five HUs and one district-level team during the intervention (June 2011–February 2012) and five follow-up months.

**Results:**

Workshops and team meetings had a 95% attendance rate. All teams gained RI system knowledge and implemented changes to address locally identified problems. Specific changes included: RI register implementation and expanded use, Child Health Card provision and monitoring, staff cross-training, staffing pattern changes, predictable outreach schedules, and health system leader—community leader meetings. Several RI system barriers prevalent across Masaka District (e.g., lack of backup HU gas cylinders, inadequate outreach transportation, and village health team underutilization) were successfully addressed. Three of five HUs significantly increased the vaccines administered. All improvements were sustained 5 months post-intervention. External evaluation validated the findings of high levels of participant engagement, empowerment to make change, and willingness to sustain improvements.

**Conclusions:**

The Microsystems Quality Improvement Approach is a comprehensive approach, grounded in systems thinking, and coupled with intensive coaching. It provides a robust framework for engaging teams in the development of unique local solutions that strengthen RI systems in resource poor settings. The sustained improvements in local RI systems from this study provide evidence that this approach may be an effective framework for enhancing the WHO’s Reaching Every District (RED) immunization strategy.

## Background

“Immunization averts an estimated two to three million deaths every year from diphtheria, tetanus, pertussis (whooping cough), and measles [1]”. However, one in five children who die before the age of 5 still lose their lives to vaccine-preventable diseases [2]. In 2012, 22.6 million children below 1 year of age were not protected against DPT3 (a proxy measure for full immunization coverage) and more than 70% of these children lived in ten developing countries including Uganda [3].

For the past 30 years, developing countries have worked to increase immunization coverage by building the infrastructure to support vaccination procurement and delivery and have relied on campaigns (child health days, national immunization days) to increase coverage rates more rapidly. While these efforts have resulted in higher and increasing rates of immunization “the proportion of the world’s children who receive recommended vaccines has remained steady for the past three years and has stagnated at about 80% DPT3 coverage” [1]. Uganda, specifically, has accomplished exemplary work focused on enhancing its routine immunization (RI) system function [4]. For example, Uganda has (a) developed district-level strategies for improvement with the World Health Organization (WHO) and partners [5], (b) participated in evaluation studies [6-9], (c) developed a training manual [4] for operational-level staff which incorporates the Reaching Every District (RED) strategy [10,11], (d) launched RED in 2003, and (e) as evidenced by the Uganda National Expanded Program on Immunization (UNEPI) multi-year plan, developed numerous strategies to sustain immunization rates when they are high and improve them when they are low [12]. However, while Uganda’s success in reaching high levels of DPT3 immunization coverage is commendable, improving rapidly from 9% in 1980 to a high of 82% in 2011, like other developing countries, its rates have stagnated around 80% (2009–2012—the past 4 years for which WHO data are available) [13].

The Africa Routine Immunization Systems Essentials-System Innovation (ARISE-SI) was designed as a proof-of-concept study to articulate an approach to systems change that addresses the pressing issue of immunization rate stagnation. This study sought to develop capacity among local community-based RI frontline workers for problem-solving resulting in innovative solutions to strengthen RI systems immediately and in the future. ARISE-SI is based on the assumption that meeting the global WHO goal of 90% national coverage for DPT3 and 80% coverage within every national district [14] requires the development of innovative approaches that take local context into account to link children to immunization services [5,15-18]. Efforts should target children from (a) peri-urban areas that do not fully utilize accessible services; (b) rural and urban populations with access to services, but who drop out of care; (c) remote rural populations with poor access to services; and (d) marginalized groups and sects [17].

Uganda’s commitment to reaching the WHO goal of 90% DPT3 coverage made it a prime site for our research initiative. ARISE-SI was sponsored by the Bill & Melinda Gates Foundation and realized through a partnership between Dartmouth College, JSI Research & Training Institute, Inc. (JSI), Makerere University School of Public Health (MUSPH), and the UNEPI, Ministry of Health (MoH).

## Methods

### Context: the microsystems quality improvement approach

The Dartmouth Institute for Health Policy and Clinical Practice pioneered the development of improvement science as it applies to health systems [19-34]. This knowledge is encapsulated in the Microsystems Quality Improvement Approach, a comprehensive approach to quality improvement (QI) practice grounded in systems thinking and coupled with intensive coaching. The approach is derived from the concept of microunits [28] and their functioning within complex systems adaptive to environmental changes [24,35-37,19].

The Microsystems Approach promotes the identification of the place in a system called the front line where the essential work actually happens. In ARISE-SI, this is where children get immunized. The approach further underscores the need to identify higher level systems (i.e., in Masaka, the District and UNEPI) that interface with the front line to facilitate work and promote achieving desired outcomes. QI methods, processes, and tools (e.g., flowcharts, data collection and display over time, small tests of change, and reflection) are applied at the appropriate system levels to encourage synergistic work toward common goals. Finally, the approach encourages teams to use data to identify system barriers and apply problem-solving techniques to develop locally appropriate changes. This differs from other approaches that provide a predetermined change package for improvement [38]. The Microsystems Approach has been successfully implemented in hospitals and ambulatory care settings in the US, Canada [39-49], and in developing countries, e.g., Kosovo [50,51]. To our knowledge, ARISE-SI is the first application of the Microsystems Approach focused on RI.

**Figure 1:**
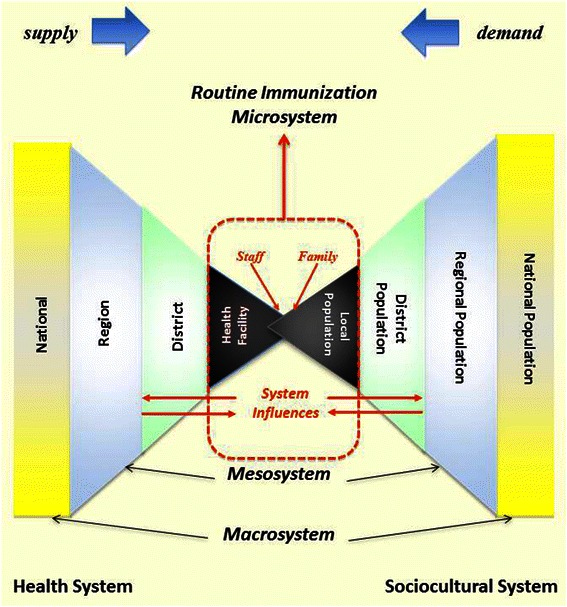
**Microsystems approach systems strengthening model.** The conceptual model for the ARISE-SI included a representation of the health system (left triangle) and the sociocultural system (right triangle). Both triangles are segmented, representing smaller aspects of each system as one moves inward in the diagram. In the center, the two triangles overlap. This represents the inextricable link between the HU and the community it serves. Together, these two segments comprise the microsystem for routine immunization.

The conceptual model that informed the design and implementation of ARISE-SI is illustrated by the “two triangle” System Strengthening Model (Figure 1). The model is based on the ARISE-SI team’s initial understanding of the RI system and sociocultural structures in Uganda.

The left triangle represents the Ugandan RI system (hospitals, clinics, outreach services). The smallest, most basic aspect is the health unit (HU). The right triangle represents the civic system; the smallest, most basic aspect is the community. The model demonstrates the importance of the linkages, and the influences of each level of system on other levels, i.e., microsystems, are embedded and function within mesosystems that are, in turn, embedded and function within a macrosystem. Thus, while the ARISE-SI intervention was primarily focused at the microsystem level (i.e., the point where a caregiver, with child, comes together with the HU staff to receive the service of immunization), the intervention concurrently included and incorporated input and context from the mesosystem (district leadership) and the macrosystem (UNEPI). In addition, it illustrates that the “service” of immunization happens within a community-HU dyad (the point where the triangles overlap) that functions within the context of cultural and socioeconomic factors as well as the dynamics of demand (people wanting services) and supply (the service is available).

### Context: Uganda’s routine immunization system

Routine immunizations are delivered through Uganda’s hierarchical health system and are not mandated by law or policy. UNEPI (macrosystem level) establishes national policy and procedures, budgets, training programs, and analyzes and disseminates RI data collected by the MoH. National Medical Stores manages all cold chain logistics for UNEPI. Each district (mesosystem level) provides education, training, supervision, and oversight of health units (microsystem level) that provide immunizations to children in clinics and outreach sites and send data back to the district where coverage is monitored.

### Study design

ARISE-SI was conducted from January 2011–June 2012. The project was a longitudinal study of QI teams that worked to improve the local RI system. In addition, the teams worked collaboratively with one another to maximize and accelerate their learning about change and improvement. The individual teams and collaborative of teams were supported by coaching. The Committee for Protection of Human Subjects at Dartmouth College in the USA and the Institutional Review Board of Makerere University School of Public Health in Uganda approved this research. All participants completed a consent form indicating understanding of the purpose of the study and willingness to participate.

### Setting and site selection

Masaka District was identified by UNEPI and the ARISE-SI team as an appropriate setting in rural and semi-urban Uganda for the study due to its (a) known high rates of immunization coverage, (b) identified leaders, (c) interest in system improvement, and (d) lack of potentially conflicting projects. Five HUs (Bukeeri, Butende, Kiyumba, Kyannamukaka, and Masaka Municipal Council) were enrolled into the study. These HUs represented the full range of governmental service-level designations HU-II to HU-IV (HU-II provides RI services only; HU-IVs are full-service clinical sites with operating theater), and each served populations of unreached children. Butende represented a non-governmental HU. Criteria for selection included that the HU (a) provided at least 250 doses of DPT1 in the previous year (a proxy measure of access to immunization services and of patient volume in the catchment area [4]), (b) was accessible by car from Masaka City, (c) had adequate staffing and management to support RI, (d) had strong relationships with at least one village health team (VHT) member (i.e., community elected volunteer residents designated as HU1 by UNEPI—the point of interface between the health system and community), and (e) assessment through site visits by ARISE-SI faculty.

### QI teams

Each HU formed a four- to seven-member core QI team including the Officer-in-Charge (usually a Clinical Officer), the staff person responsible for RI (RI focal person), a staff member trained in Health Management Information Systems (HMIS), and at least one VHT member. At the request of the Masaka District Health Officer, a district QI team including the District Health Inspector, HMIS Officer, Senior Nursing Officer, Health Educator, and Cold Chain Officer participated in the study.

### Intervention

The intervention consisted of three main components: advance preparations, an action learning collaborative, and coaching of QI teams.

#### Advance preparations

The goals of the advance preparations were to (a) introduce ARISE-SI to HU staff and community members and obtain their commitment to the intervention; (b) establish QI teams; (c) increase the QI teams’ knowledge of the supply and demand sides of their RI microsystem; and (d) prepare the teams for the first meeting of the action learning collaborative. A local assessment was completed by each QI team in partnership with community stakeholders. Using local data, the team summarized the HU RI system’s function based on five themes: people, personnel, process, purpose, and patterns [19]. To obtain more information about enablers and barriers, ARISE-SI faculty conducted focus group discussions with community members. (Interview guide is available by request.)

#### Action learning collaborative

An action learning collaborative was the vehicle for implementing the Microsystems Approach. Members of the collaborative were the five HU teams, the Masaka District team, the UNEPI Training Director, and the ARISE-SI faculty and coach. The collaborative brought the six QI teams together to study their RI system from multiple perspectives and create a higher level “system” awareness of problems that could be improved. Activities of the collaborative included teaching the principles and practice of systems thinking and QI, providing technical support and training specific to RI, fostering shared learning and communication within, between, and across team functional roles and systems, and training the coach to mentor the teams through a QI project [52-58]. The collaborative ran for the entire 9-month study intervention period.

**Figure 2:**
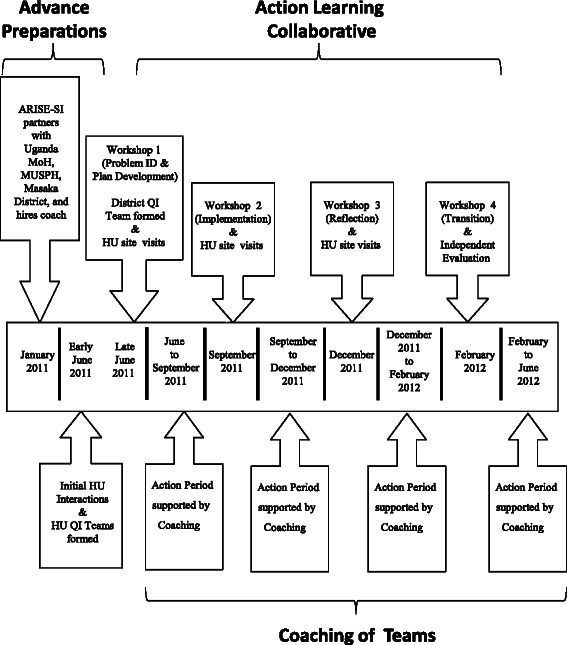
**ARISE-SI intervention timeline.** The ARISE-SI Project began with advance preparation to establish Ugandan project partners in January 2011. Advance preparations continued with initial HU assessments and formation of HU QI teams in June 2011. The District QI team was formed in June 2011 at Workshop 1 of the action learning collaborative. The collaborative included sequential action periods, HU site visits, and workshops which continued through February 2012. Coaching provided support to QI teams between collaborative workshops.

The specific activities of the collaborative were workshops, on-site HU visits, and local QI team meetings between workshops (action periods) (Figure 2). QI teams came together to attend four 2- or 3-day workshops in Masaka City (June, Sept, Dec 2011, and Feb 2012). Themes for the workshops in sequential order were problem identification and improvement plan development, improvement plan implementation, reflection on the improvement process, and transition to local ownership. During each workshop, Dartmouth and MUSPH faculty, the coach, and the UNEPI Director of Training adapted and taught the Dartmouth Microsystems Curriculum [19] and provided technical assistance and training on RI and cold chain maintenance [5,59,10,4,11]. The UNEPI Director of Training and the District Health Inspector addressed technical issues raised by the QI teams and traveled with the faculty and coach to HUs, mentoring teams during educational site visits held before or after each workshop. Interactive and participatory teaching methods engaged participants with each other, the curriculum, and with the issues they were working to improve.

Workshops and HU site visits guided QI team planning, implementation, and measurement. Teams were challenged to develop system-strengthening solutions using existing staff and local budgets. Each team identified a unique aim and designed their improvement project based on data generated from their baseline assessment, immunization data, and understanding of local context and culture. Implementation and evaluation of improvements were accomplished through plan-do-study-act (PDSA) cycles of improvement [60]. Teams were taught to use run charts to monitor the changes in numbers of immunizations provided at HUs and outreach sites. Reliable denominator data were not available for geographic areas smaller than Masaka District, thus rates of coverage were not considered for benchmarking. At each workshop, QI teams presented their work to each other in a facilitated forum. Team members were encouraged to take on the roles of learners and teachers; thus, questions that arose were addressed by other participants, the District, UNEPI, or ARISE-SI faculty. Grant resources funded the time and travel of the faculty and coach, reimbursed participants for workshop time, lodging, and travel, and funded participants’ per diem expenses at HU educations sessions.

#### Coaching

The coach provided technical support, project management, and mentoring of QI teams. He was mentored by a US-based ARISE-SI faculty member through bi-weekly phone/SKYPE calls and quarterly in-person support in Uganda. The MUSPH faculty, UNEPI, and coach attended a “coach the coach” workshop at Dartmouth to learn the principles and methods of QI and coaching. Support for the coach’s time (one full-time salary) as well as his travel to the HUs and the collaborative workshops was provided through ARISE-SI funding.

### Data sources

Quantitative and qualitative data were collected during each aspect of the learning collaborative (workshops, site visits, and action periods). Data from these multiple sources supported on-going evaluation of the intervention’s fidelity, improvement processes, and outcomes. These data also helped the researchers identify and assess contextual factors of each level of the RI system at each site over time [61-65]. The major data collection activities during ARISE-SI are described in Table 1. These included (a) assessment of immunization doses administered to children, (b) initial assessment by each HU of it RI system functioning, (c) data presented by QI teams as well as workshop evaluations, (d) field notes and observations by the coach during QI team meetings, and (e) an external evaluation of the project completed by researchers not affiliated with the study.

**Table 1:** **Detailed description of data collection including purpose, sources and methods, and measures for each ARISE-SI activity**

Activity	Purpose	Source and method	Measures
Assessment of immunization doses	• Establish baseline and monitor trends associated with system improvements	• Usual administrative data reported from the HUs to the District and from the District to UNEPI^a^	• Number of DPT1 and DPT3 doses^b^: DPT1 static, DPT1 outreach, DPT3 static, DPT3 outreach
Initial assessment at HUs (June 2011)	• Develop improvement teams • Gain in-depth understanding of each HU’s context related to RI	• Caregiver focus groups • 5 Ps—purpose, mapping hard to reach people, personnel, process flow charts, patterns • Introduce HUs to meeting skills	• Microsystem components • Barriers and enablers to RI
4 Participatory workshops (attended by five HU and one district QI team: June 2011, September 2011, December 2011, February 2012)	• Problem identification • Improvement plan development • Implementation of improvement plan • Reflection on improvement process • Transition to local ownership	• Pre-workshop participant information survey • Before and after action reviews • Observation • Workshop evaluation	• Specific workshop objectives • Pre-intervention baseline: QI knowledge and work environment • Interest and acceptance of Microsystems Approach • Knowledge, skills, and abilities related to Microsystem Approach • RI knowledge • Barriers/enablers to RI • Team and collaboration skills • Ability to work across systems
QI team coaching (June 2011–February 2012 between workshops)	• Support progression of QI teams’ improvement work • Foster linkages between the district and HU staff and community	• PDSA tracking matrix • Run charts • Attendance roster • Meeting minutes • Coach’s reflective journal • Technical assistance from coach	• Implementation of improvement plans • Consistency of team participation in meetings • Emerging leadership • Group function • Meeting skills • Coach’s role development
Evaluation by researchers external to project^c^ (February 2012)	• Validation of findings	• Focus groups • Survey	• In-person meetings of all workshop participants using structured interview guide • Written questionnaire completed individually by workshop participants

^a^RI data were provided to us by the District Health Inspector.

^b^RI data were collected and recorded in the usual way by the HUs throughout the intervention period and were transposed in an Excel spreadsheet by the District Health Inspector and the Coach.

^c^Data were collected by Ugandan researchers, guided and analyzed by Center for Program Design and Evaluation at Dartmouth College.

### Data analysis

Triangulation of data assured an in-depth assessment of the intervention [66]. Data were analyzed using a mixed-methods approach. Initially, qualitative and quantitative data were analyzed separately then examined together. Research findings were validated by external evaluation.

Quantitative data related to attendance and evaluation of workshops, improvement team meetings, and associated immunization data were summarized. Qualitative data were analyzed using an iterative coding and data reduction process [66,67] in which a preliminary coding scheme was developed based on grounded theory technique [68] and then analyzed using NVIVO 9, applying a process of continual comparison of findings over time. Emerging themes about learning and application of QI skills by the improvement teams, as well as before and after action reviews [69,70] and workshop evaluations were used by ARISE-SI faculty to refine the intervention (workshop content, teaching methods, coaching approach) as it was implemented. Counts of doses of DPT1 and DPT3 vaccines administered at each HU clinic and outreach sites were aggregated monthly. DPT1 and DPT3 counts were used as proxies for access and coverage, respectively [71,4,5]. A two-tailed, unpaired *t*-test was used to compare the average monthly immunization doses administered at baseline (June 2010 to May 2011) with the intervention and follow-up periods (June 2011 through May 2012). In addition, independent Ugandan researchers conducted an external evaluation of the intervention (February 2012) using a mixed-methods triangulation design [72]. The evaluators conducted five focus groups and administered a comprehensive written questionnaire to participants prior to the final workshop session in the absence of project faculty.

## Results

### Intervention fidelity

All components of ARISE-SI were implemented successfully during the planned timeline. The four workshops that formed the educational basis of the action learning collaborative were successfully completed (June 2011–February 2012). Participant evaluations indicated that workshop objectives were met. External evaluation demonstrated that participants rated the quality of the teaching, coaching, and overall project highly. For example, respondents indicated that the teaching methods and activities helped them learn about QI, meeting skills, coaching, and setting team-specific aims. Mean ratings on these teaching activities ranged from 4.2 ± 0.67 to 4.6 ± 0.5 on a 5-point scale with “5” being the highest rating. (Full details are provided in the project report available online [73].)

### Participation

**Figure 3:**
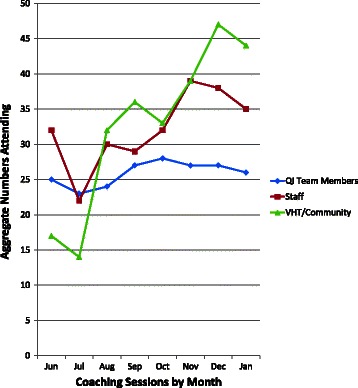
**Numbers of participants attending monthly HU coaching meetings.** The coach held monthly meetings at each of the HUs beginning in June 2011 and continuing through January 2012. Participants for all HU meetings combined are shown for QI team members (blue diamonds), HU staff (red squares), and VHT/community members (tan triangles).

The four workshops and associated education sessions were well attended. HU and district QI team members participated in virtually every workshop (95% attendance). During action periods, the coach met monthly with each QI team helping them complete assignments from the previous workshop. In all, he held eight meetings at each HU. Meeting participation combined across HUs was excellent: the five HU QI team members remained stable (23–28 persons, median = 27, SD = 1.62); numbers of other HU staff attending ranged from 22–39 persons; and participation among community members increased from 14 persons in June 2011 to 44 in December 2011 (median = 34.5, SD = 11.07) (Figure 3).

### Local solutions to long-term problems

QI teams developed unique, HU-specific project aims, designed changes and associated process measures, implemented two PDSA cycles of change, and evaluated outcomes. As illustrative examples of the successes achieved by teams, we highlight three major barriers to RI system function that were addressed during the study and were recognized by UNEPI as prevalent across Masaka District: (a) lack of a backup supply of gas cylinders at HUs, (b) inadequate transport from HUs to outreach sites, and (c) underutilization of VHTs [12]. The local solutions to these long-term problems are summarized in the case studies below.

#### The story of the gas cylinder

Gas cylinders are used in most Masaka District HUs to power refrigerators to keep vaccines at the right temperature to maintain potency. The District Health Inspector summarized the implications of not having a backup cylinder in a short brief to USAID: “For at least the past ten years there has been only one gas cylinder in the HUs in Masaka instead of two. This shortage affects the cold chain. For example, during the time it takes to refill the one existing cylinder (up to one month), vaccines may be improperly stored with potency compromised, may be wasted, or routine services may be interrupted; all resulting in lower immunization coverage [74]”.

To address this problem, the district QI team developed the specific aim to obtain a second gas cylinder for every HU in Masaka District (their inventory revealed that 22 of the 33 units had gas powered refrigerators). The team used QI tools to identify points of leverage for action. Their improvement plan proposed a reallocation of funds from the District Primary Health Care budget to procure additional gas cylinders, provided data affirming that a reallocation of funds would not affect other primary care services, and included a detailed proposal for tracking and monitoring the flow of gas cylinders through a more rigorous inventory tracking and control process which included financial responsibility for the HU if a cylinder was lost or stolen. The District Health Officer approved and helped negotiate the plan with the District Health Committee. As a result of this improvement initiative, the District Health Office procured 22 gas cylinders in December 2012, supplied each HU using gas with a backup cylinder, and integrated inventory control into their quarterly support supervision process. As of May 2014, no gas cylinders have gone missing and the inventory control process is intact and functioning (verbal communication with the ARISE-SI coach).

#### The story of the motorcycle

Bukeeri (HU-III) provides RI at the HU and at four outreach sites for the 10,000 people in its service area. Many roads in Bukeeri are gravel, some are dirt. UNEPI provides each HU-III a motorcycle for transporting immunizations to outreach sites. Four months prior to ARISE-SI, the Bukeeri motorcycle broke down. Since there was no money to repair the motorcycle, Bukeeri closed its outreaches. Thus, vaccines were unavailable to those who were not able to get to the HU.

During the first ARISE-SI workshop, the Bukeeri team learned from their colleagues that two other HUs had motorcycles that were out-of-service. However, these HUs had kept their outreaches open by reallocating funds from the “fuel” for the motorcycle line item in their Primary Health Care Funds to hire a motorcycle driver to transport staff and immunizations to outreaches. Building on this new knowledge, the Bukeeri team developed an improvement plan to implement a similar solution. In addition, based on information from their own baseline assessment, Bukeeri’s plan included a communication strategy to negotiate dates and times of outreaches with VHTs and villagers. As a result, the four outreaches were reopened in Bukeeri’s service area (July 2011) and were well attended. As of May 2014, the four outreaches remain open (verbal communication with ARISE-SI coach).

#### The story of the VHTs

The in-charge and staff at Kyannamukaka (HU-IV) have many duties including providing primary care, maternal child health services, deliveries, inpatient care, and RI. VHTs, although trained to coach families on RI, were not well integrated within the RI system function. Thus, the Kyannamukaka QI team focused their improvement plan on capacity building of VHTs. Planned changes included: (a) training VHTs to read Child Health Cards, (b) supporting VHTs in visiting 25 homesteads and checking each child’s immunization record and status, (c) having VHTs include phone contacts of caregivers into the HU registry to facilitate follow-up of children, (d) developing a duplicate HU registry so these data could be taken to the field for VHT use, (e) and including VHTs at regular meetings.

Sixty VHTs were trained to read Child Health Cards. The QI team held meetings with these newly trained VHTs and developed plans for home visits. The VHTs provided the HU with lists of the names of homesteads visited during September–January with children under 1 year of age. Through this process, they identified three children who had not had measle vaccinations and referred them to the HU. In addition, families in two villages who had not previously had their children immunized are using these services and receiving vaccines.

### Changes and associated improvements in RI system function

#### Enablers and barriers identified and addressed through QI team projects

Thirty enablers and barriers to RI were identified by HU staff and community members at baseline. These factors are commonly described as being important to RI system strengthening [12]. Twenty-one of these factors were addressed through the QI teams’ projects, enhancing enablers and reducing barriers. (*Addressed through QI team projects)

### Enablers

Immunizations provided at no cost*

Accessible services*

Approachable, competent staff*

Supplies in stock (vaccines, Child Health Cards, gas cylinders)*

Reliable schedules*

Community involvement*

Leadership*

Monthly meetings to discuss unreached*

Integrated outreach*

Active staff (RI focal person)*

Effective and timely reporting*

Public messaging, mobilizing campaigns

Schools require immunization for enrollment

Mothers of child-bearing age are immunized

### Barriers

Inconsistent follow up*

Outreach unavailable*

Lack of awareness about RI*

Lack of resources (stockouts)*

Long wait lines*

Insensitive staff attitudes*

Cultural beliefs*

Transportation*

Unaware of schedules*

Mothers miss clinic*

Transient population

Staff absent

VHT too busy

No allowance for VHT

History of sickness or death from immunization

Family issues

#### Changes initiated by QI teams and associated improvements in RI system function

QI teams initiated changes focused on improving the internal processes associated with RI service delivery as well as improving communication, relationships, and education associated with RI. All HUs worked to engage or strengthen VHT involvement in their QI team’s efforts to improve internal system delivery as well as external engagement with community leaders and families. As illustrated in Table 2, process changes resulted in improvements to specific aspects of RI system function (e.g., increased numbers of VHTs making home visits to monitor Child Health Cards and encourage families to go to HU or outreach sites for RI, increased numbers of VHTs trained to work with families and clinic staff to engage families and community to obtain RI for their children, decreased wait time at HU for RI services, and increased number of meetings between the HU In-Charge and community leadership to promote RI services). In three of five HUs, process changes were associated with significant increases in DPT doses administered by the HU during the intervention and follow-up period. Specifically, there were eight significant increases in the number of DPT doses provided to children during intervention and follow-up. Four of the increases (both DPT3 and DPT1 at the HU and at outreach sites) occurred in Bukeeri HU where closed outreach sites were opened and VHTs were mobilized to engage families and caregivers to immunize their children.

**Table 2:** **Description of changes initiated by QI teams and associated outcomes including data on number of DPT doses administered**

Improvement team	Examples of changes initiated (June 2011–Feb 2012)	Associated outcomes at intervention end (Feb 2012)	Average monthly number of doses of DPT antigens: comparing baseline with project implementation and follow-up periods (June 2010–May 2012)				
			Antigen	HU type^a^	BL avg^b^	PIF avg^c^	Sig^d^
Bukeeri	Reallocated existing budget to pay a local motorcycle driver to take staff to outreach sites to provide RI services; established partnerships between staff and VHTs to improve access to population; met with and engaged religious leaders	Four outreach sites opened and providing RI on a regular basis at times negotiated with community; VHTs mobilized mothers and visiting households to check status of child health cards; tally sheets and registration forms developed to monitor outreach	DPT3	Static	23.3	31.0	*p* = 0.038
			DPT3	OR	2.6	33.7	*p* < 0.001
			DPT1	Static	25.2	33.3	*p* = 0.036
			DPT1	OR	3.5	28.3	*p* < 0.001
Butende	Incorporated VHTs into data collection and improvement process; changed existing staffing pattern to increase RI staff from one to two on RI days; improved staff arrival time at outreaches; directly involved VHTs in mobilizing families; met with religious leaders	VHTs now provide input to improvement process; VHTs making home visits to “difficult areas”; staff arrival time at outreaches becoming more consistent; in-charge actively working with religious leaders	DPT3	Static	4.8	8.7	*p* = 0.008
			DPT3	OR	29.4	28.8	NS
			DPT1	Static	4.8	9.3	*p* = 0.002
			DPT1	OR	30.4	35.2	NS
Kiyumba	Cross-trained 17 staff on RI techniques; put two vaccinators on duty on days when RIs are administered; reorganized process of RI; expanded involvement of VHTs	Decreased wait time for RI to less than 1 hour from 80% of clients to 20%; VHTs making home visits and identifying unimmunized children	DPT3	Static	28.7	22.1	NS
			DPT3	OR	20.5	21.6	NS
			DPT1	Static	22.7	25.7	NS
			DPT1	OR	18.6	22.7	NS
Kyannamukaka	Ensured that all children receiving services had a child health card; implemented use of registers which included phone numbers, home visits by VHTs, and plan for staff to f/u with caregivers using phone	VHTs visited at least 25 households; have held village meetings; engaged other stakeholders in learning about RI, are referring children to HU; 60 VHTs have been trained by staff; one outreach site has become a static site	DPT3	Static	22.8	20.3	NS
			DPT3	OR	34.4	33.8	NS
			DPT1	Static	23.4	20.4	NS
			DPT1	OR	31.3	30.3	NS
MMC	Increased the number of RI staff to three on most days of the week and to two on outreach days; VHTs were to visit 25 homes, screen all children at static unit for RI status	Improved communication among caregivers, VHTs and staff; developed system for tracking home visits; VHTs identify cases of resistant families and successfully got them to RI; HU working with District leadership to engage other resistant families	DPT3	Static	39.1	65.3	*p* < 0.001
			DPT3	OR	11.7	13.0	NS
			DPT1	Static	47.0	69.7	*p* = 0.001
			DPT1	OR	12.8	10.4	NS
District health team	Reallocated existing primary care budget to accommodate the purchase of 22 gas cylinders; advocated for purchase by showing no unintended consequences to other services; developed a tracking system to monitor location and use of cylinders	22 gas cylinders purchased and distributed to HUs with tracking system in place	NA	NA	NA	NA	NA

*NA* not applicable, as the District Health Team did not directly engage in administration of vaccinations. Their efforts supported the processes for vaccine delivery and storage.

^a^HU type: Static units are the actual physical location of the health unit building. Outreach sites (OR) are places in surrounding villages where immunizations are routinely provided on scheduled days during the month.

^b^BL avg: Baseline average number of antigens administered from June 2010 to May 2011.

^c^PIF avg: Project implementation and follow-up average number of antigens administered during project intervention and follow-up periods from June 2011 to May 2012.

^d^Sig: significance of changes noted: two-tailed unpaired *t*-test comparing BL and PIF periods; NS means that *p* > 0.05 in antigens administered during the life of the project.

## Discussion

This study demonstrates that the Microsystems Quality Improvement Approach provides a robust framework for developing local solutions and improvements to strengthen local RI systems in resource poor settings. Incorporating systems thinking, principles and practice of QI, and coaching inspires system-wide learning and opportunities to build capacity and ownership of system processes and outcomes among front-line workers. In the 9-month study period, participants gained a working knowledge of the local RI systems in Masaka District. Participants leveraged, implemented, and monitored changes, and in some cases, sustained improvements (e.g., higher than average DPT doses for at least 5 months after the intervention period). In addition, back-up gas cylinders remain in place in all Masaka HUs, Bukeeri’s outreaches remain open, and VHTs are still more engaged in RI than they had been 2 years after the intervention period (per communication from Ugandan coach).

Previous approaches to RI system strengthening have focused on targeted aspects of a system such as: increasing the supply of immunizations and improving management practices [11,75-79], changing practices at specific sites [76,79,11,80], bringing immunizations closer to communities [81-83], increasing demand for immunizations by using information dissemination [84,82,85-88], or providing incentives to caregivers [89,87]. The Microsystems Approach focuses on building system capacity for on-going assessment, problem-solving, and evaluation. The approach accomplishes this by (a) promoting systems thinking and active problem-solving within and across multiple levels of a system, (b) developing multidisciplinary QI teams, using tools and training that foster team ownership of a system and its outcomes, (c) providing a non-prescriptive educational curriculum focused on QI principles and systems thinking that incorporates knowledge of locally identified barriers and enablers, and (d) in the case of ARISE-SI, expecting that teams could design, implement, test, and refine solutions using existing staff and within current budgetary constraints. ARISE-SI likely achieved its effects through these unique factors that provided a platform for local problem-solving.

**Figure 4:**
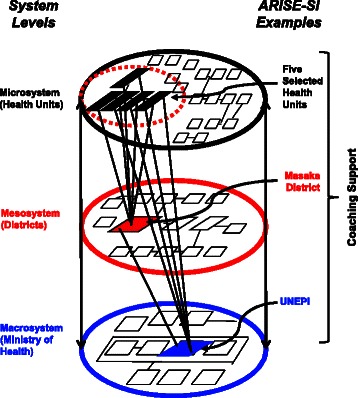
**Interactions among different levels of systems associated with ARISE-SI.** The ARISE-SI project engaged three levels of the RI system—HUs (microsystem), Masaka District (mesosystem), and UNEPI (macrosystem). The project brought representatives from each system level together for all project activities. The coach facilitated communication and interactions both within and across the three system levels.

The action learning collaborative intentionally brought key leaders from three distinct levels of the RI system together to develop a shared understanding of system complexity and to promote innovation in problem-solving based on shared knowledge. The educational tools and action-learning process of the workshops, as well as the development of multi-disciplinary teams, were designed to break down the existing hierarchical and siloed approach to communication, learning, and decision-making. For example, at Kiyumba (HU-IV), one person was designated as the focal person for RI. If this person was late/absent from work, his/her role was not typically filled by other staff members, resulting in missed opportunities for immunization. To address this issue, the Kiyumba QI team cross-trained HU staff on immunization practices, giving the clinic the ability to accomplish RI at almost any patient encounter. The coach’s role was critical for enhancing communication within and across levels of systems as he met with all study participants monthly and was the one “constant” of the intervention and in all communications. Figure 4 illustrates the within, across, and up and down communication, learning, and decision-making that was promoted during the intervention.

The QI teams’ projects were based on assessment of baseline data. The workshops provided a safe space for HU members to ask questions directly of district and UNEPI leaders. This resulted in “just-in-time” clarification of issues related to RI policies and practices that promoted local problem-solving. For example, the District Health Office’s approval of the reallocation of the “gas” line item in their budget gave permission to Bukeeri (HU-III) to implement this as a change strategy. Additionally, the development of QI knowledge and skills may have promoted participants’ self-efficacy and improved team member status within their HU and community. Team members benefited from learning new skills that they could apply to other problems. For example, the District Health Inspector applied this approach with his sanitation team before the ARISE-SI workshops were completed.

Furthermore, the Microsystems educational curriculum and approach [90] may have accelerated the participants’ abilities to brainstorm freely about problems and solutions and learn how issues were being addressed at other HUs. For example, the teams’ presentations of their baseline data highlighted that the lack of a second gas cylinder affected their ability to provide quality RI services. The district team’s prioritization of this issue in their improvement project validated the workshop discussions. Likewise, the Bukeeri (HU-III) team learned how to use their own budget to get important needs met and also learned that other HU teams and district leaders are important resources for information, guidance, and problem-solving.

UNEPI identifies the VHT as the first level of contact for RI and as essential to system function [91]. During the baseline assessment, information emerged that VHTs were disconnected from HUs and unclear about their RI role and function. In an effort to maximize this resource, the intervention encouraged participation by VHTs on QI teams. Several teams enhanced the training of VHTs, and all HU teams included VHTs in their QI plans. These efforts may have created more demand for RI services from families. Participation in the project may have harnessed the VHTs’ desire to provide excellent care to those that they served, thereby contributing to their increasing participation at HU QI meetings. On the other hand, it is also possible that the growing participation of VHTs was in part associated with the per diem paid for every meeting VHTs attended, as such payments are of great importance in developing countries.

All teams demonstrated success in applying QI methods through implementation of cycles of change to make system improvements. Analyses of RI data were required to assess progress toward each HUs improvement aim. As HUs learned more about their own RI system, it became increasingly important to them to understand RI data stratified by where immunizations were provided—at the HU itself or at outreach sites. RI data were discussed concurrently with process and intermediate outcome data collected throughout improvement efforts. HUs reported that using their own data in this transparent way (displaying data over time on graphs, stratifying RI data based on service delivery sites, and sharing data with other HUs and the DHT) helped promote engagement and sustain the improvement work.

Improvements implemented during ARISE-SI were locally derived and innovative within the context in which they occurred. The heterogeneity in improvement in the number of DPT1 and DPT3 immunizations administered during the initiative should be considered in the context of the changes chosen and implemented by each HU. For example, Bukeeri (HU-III) opened outreach units that had been closed for several months and evidenced rapid, significant improvement in doses of immunizations administered in outreach units. Kiyumba (HU-IV), on the other hand, focused on reducing waiting times for mothers at the HU. Thus, while Kiyumba successfully met its goal, it experienced no significant changes in doses of DPT provided.

One could argue that the improvements to the RI system implemented during ARISE-SI were not innovative. However, for purposes of this study, innovation was defined as creative, local problem-solving [92]. To this end, the six QI teams designed innovative solutions that when implemented, immediately began to strengthen local RI systems and function. Some of the changes made by the QI teams might have occurred without ARISE-SI. However, the timing of all improvements (e.g., opening of outreach clinics, ensuring HUs have two gas cylinders, increasing training and mobilization of VHTs, reducing wait times at HUs, enhancing use of immunization cards) followed the initiation of the intervention, as did the upward trend in numbers of vaccines administered. Furthermore, problems such as closed outreach clinics and shortage of gas cylinders had existed for months/years prior to ARISE-SI. Thus, it seems highly likely that these improvements were at least in part related to the intervention.

The small ARISE-SI sample size limited the ability to utilize immunization data for analyses of rates. However, the problem of calculating coverage rates exists for any intervention in a small area of analysis. The use of proxy measures (counts of DPT1 and DPT3 doses administered to access trends of change) provided some assurance that the changes to the system resulted in improved outcomes. In addition, while the absolute number of QI teams was small, they were selected to represent every level/type of HU in Masaka District. The engagement of a district-level QI team provided the possibility of effecting change that could reach beyond the five HUs [90]. It remains possible that part of the intervention’s success was related to more attention than usual focused on RI and that this induced team members to feel more accountable to the work. Additional research would be required to disentangle this question.

Although initial external support to develop, train, and maintain the QI teams was resource intensive, this support was necessary since Microsystems Approach expertise did not exist in Uganda before the intervention. It is feasible that the key elements of this intervention could be adapted by UNEPI and MUSPH and be embedded within the Ugandan health care system training and management infrastructure so that these elements could be implemented on a larger scale going forward. For example, a rigorous QI process and application of systems thinking could be built into the proposed infrastructure of RED. In 2002, the WHO and its partners developed and began implementation of RED [93,10,11] with the aim of strengthening RI services by focusing on the district level for immunization service delivery [5,16]. RED has five core components: (a) planning and management of resources, (b) reaching target populations, (c) linking services with communities, (d) supportive supervision, and (e) monitoring for action specifically within the context of a microplanning process [16].

Recent studies in Uganda (e.g., The EPI Review 2010 and Effective Vaccine Management Assessment (EVMA) 2011) showed operational inadequacies of the immunization system related to the supportive supervision and advocacy and communication aspects of RED [12]. The initial assessment of each HU in ARISE-SI corroborated these findings as well as other challenges in Masaka District’s RI system. Specifically, HU staff noted difficulty in identifying and prioritizing barriers to their RI system as they had no approach for doing this. Similarly, staff lacked an approach for developing and implementing microplans. While the staff knew the population they served well, they were not reaching out to the community for input on their immunization processes. VHTs were available and willing to help HUs but neither the HU nor the VHTs fully recognized how to maximize the VHT role in the RI system. Finally, HU staff noted having very little on-site training or opportunity to clarify issues or questions related to RI.

While RED provides for and mandates supportive supervision as well as its other components, it does not provide robust tools and training to operationalize these components. As noted in Table 3, the design of ARISE-SI incorporated a focus on both the supply and demand for immunizations as well as several of the components of RED and may provide an approach and tools to operationalize this national strategy. Specifically, ARISE-SI supported the development of HU teams that were focused on improving the RI system (these teams did not exist before ARISE-SI). ARISE-SI funded monthly in-person or phone meetings between the coach and each HU improvement team. The District Health Inspector joined the coach during all in-person HU visits. This partnership enhanced the supportive supervision component of RED in two major ways. First, funds were made available through ARISE-SI to pay staff (specifically the VHTs) the usual per diem rate for travel and food so that they could attend the HU team meetings. Most importantly, these frequent meetings created an opportunity for the ARISE-SI coach to train the District Health Inspector in the Microsystems Approach and for the District Health Inspector to train the HU teams on technical aspects of RI.

**Table 3:** **Reaching Every District (RED) components with associated description mapped to ARISE-SI advance preparation findings and activities**

Reaching Every District (RED)		ARISE-SI	
Component	Description	Advance preparation findings	Activities
1. PLANNING AND MANAGEMENT OF RESOURCES: better management of human and financial resources.	At the district and facility levels, planning should identify what resources are needed to reach all target populations in a way that can be managed well and thus maintained. Good planning involves: (a) understanding the district/health facility catchment area (situational analysis); (b) prioritizing problems and designing microplans that address key gaps; (c) as part of microplanning, developing a budget that realistically reflects the human, material and financial resources available; and (d) regularly revising, updating and costing microplans to address changing needs.	• Integrated care and services: drugs draw people; lack of interest may prevent people from coming. • Record Keeping and Management: use of registers for tracking waiting times, home visits, follow-up calls, Child Health Cards. • Roles: VHT can go to homes; know roads, residents, who are immunized, provide health education. • Scheduling: waiting time important issue to mothers; reliability of schedule is important. • Staffing: HU staffing does not align with UNEPI standards; however HUs agreed that they are often able to provide services with the staff that they have. • Supplies: Child Health Cards, vaccine and gas stock-outs common across HUs. • Education and Training: VHT eager to learn; training needs include HMIS, RI-TA and QI training. • Cold Chain: Lack of affordable fuel for transport; motorcycles are in disrepair; difficult passage on roads; lack of adequate gas cylinders.	• Complete initial assessment of current state. • Agree on importance of children having Child Health Cards. • Re-allocate PHC funds to hire local taxi. • Purchase gas cylinders. • Change HU and outreach schedules to accommodate child care-givers needs. • Increase staffing on RI days. • Maximize VHT capacity for RI. • Incorporate VHT into HU QI Team. • Child Health Card used as documentation, communication. • Cross-train staff in RI. • Develop better understanding of VHT assignment and HU service area.
2. REACHING TARGET POPULATIONS — improving access to immunization services by all.	“Reaching the target populations” is a process to improve access and use of immunization and other health services in a cost-effective manner through a mix of service delivery strategies that meet the needs of target populations.	• HU staff seemed to know their populations well. • Families suggested the need for integrated services. • VHTs are trained to promote general and specific services. • Reliability of scheduling is very important to families.	• HU staff were able to draw maps of their service area and identify where services are delivered and where hard to reach persons lived. • Incorporated VHT into HU QI Team. • VHT increased home visits. • VHT educated about RI. • Staff taught VHT to read Child Health Cards. • Increased staffing on RI clinic days. • Opened outreaches. • HU adjusted hours of outreach clinic to accommodate mothers’ need for working in gardens. • Staff arrived on time at outreaches.
3. LINKING SERVICES WITH COMMUNITIES — partnering with communities to promote and deliver services.	This RED component encourages health staff to partner with communities in managing and implementing immunization and other health services. Through regular meetings, district health teams and health facility staff engage with communities to make sure that immunization and other health services are meeting their needs.	• HU management committee and community leaders involved. • Many HUs using mobilizers and VHTs. • HUs are beginning to train VHTs.	• Caregiver focus groups identified specific needs of each HU service area. • VHTs were included as members of HU QI Teams. • Staff and VHTs met with religious leaders. • VHTs were enlisted from communities with unreached, including Muslims.
4. SUPPORTIVE SUPERVISION — regular on-site teaching, feedback and follow-up with health staff.	Supportive supervision focuses on promoting quality services by periodically assessing and strengthening service providers’ skills, attitudes and working conditions. It includes regular on-site teaching, feedback and follow-up with health staff.	• HU staff had many questions regarding RI policy and practice.	• Coaching included focus on QI, use of data, display of data, education/instruction about technical aspects of RI practice. • Workshops focused on addressing identified technical information needs: overview of RI in Uganda, VHT Program, understanding RI rates, RI administration policies and included interactive sessions wherein HU teams educated one another on specific topic areas.
5. MONITORING FOR ACTION — using tools and providing feedback for continuous self- assessment and improvement.	District health teams and health facility staff need a continuous flow of information that tells them whether health services are of high quality and accessible to the target population, who is and is not being reached, whether resources are being used efficiently and whether strategies are meeting objectives. Monitoring health information involves observing, collecting, and examining program data. “Monitoring for Action” takes this one step further, by not only analyzing data but by using the data at all levels to direct the program in measuring progress, identifying areas needing specific interventions and making practical revisions to plans.	• Each HU has an assigned HMIS person on staff. • Used data for reporting to DHO (immunizations, drop outs, etc.) • HMIS persons understand how to collect, and display data. • Data are not used for assessment or tracking of improvements.	• Use of QI tools: fishbone, PDSA, Model for Improvement, Ladder of Improvement, operational definitions, data collection, data display, meeting skills. • Data collected and used for improvement: caregiver waiting times, # children w/ Child Health Cards, # homes visited by VHTs, # outreach sites open, # VHTs instructed on reading of Child Health Cards, etc. • VHT registries and patient registries as data sources. • Engaging VHTs in process of collecting data and understanding how it is used for improving RI services within their HU service areas. • HMIS instructing staff on role of data for improving their processes. • Regular meeting of HU QI Team, use of meeting skills to maximize productivity of staff and time.

ARISE-SI made a concerted effort to address the advocacy and communication aspect of RED. VHTs were included as functioning members of the new RI improvement teams. The VHTs and health unit staff worked together to design outreach strategies that enhanced communication between the VHTs, villagers, and HU staff. In addition, the District Health Inspector met directly with VHTs to describe and emphasize their key role in the RI system. The VHT numbers at the HU meetings grew during ARISE-SI which might suggest that the VHTs were more engaged with and had a better understanding of the powerful role they could play in enhancing immunization rates in their own communities.

Finally, ARISE-SI included funds to bring the five HU teams together and provided hands-on technical assistance on work flow, cold chain maintenance, and VHT training by RI technical experts (the UNEPI Director of Training and District Health Inspector), the trained coach, and QI experts from the ARISE-SI team. The coach worked to coordinate the HU team meetings within the supportive supervision infrastructure that already existed in Masaka District. To enhance sustainability of this approach, coaching expertise could be provided by district-level staff (e.g., the District Health Inspector) and QI-focused coaching could be embedded within usual district supportive supervision. Thus, implementing the Microsystems Approach concurrently with RED by engaging national (UNEPI), district, HU, and community participation may be an effective strategy for linking knowledge to practice to actualize technical RI information and for leveraging systems improvements across HU and district service areas. That said, it must be noted that ARISE-SI did not study the effects on RI system strengthening that might be achieved if similar funds as those used for this study were employed solely in support of RED supportive supervision, or for other types of supportive supervision within Masaka District. This remains an open question for future research.

In summary, this intervention fostered the development of sustainable local solutions by multidisciplinary teams across system levels. The ARISE-SI study findings prompted interest from UNEPI in embedding QI and systems thinking policy and practice into existing training and management systems. UNEPI also recognized that beyond RI, the Microsystems Approach has been proven to be applicable to a range of health issues [39-49]. Thus, building a work force capable of applying systems thinking and QI tools could enhance the broader Ugandan work of health system strengthening.

## Conclusions

This proof-of-concept study illustrates how a structured change process such as the Microsystems Approach can successfully spearhead and support system strengthening through development of local solutions to address entrenched problems within a RI system in Uganda. This approach may provide an effective framework for actualizing the WHO Reaching Every District core components. Research to apply this approach within the training, meeting, and supervisory infrastructure that already exists within the Ugandan RI system is needed to assess costs and benefits of adapting such an approach on a larger scale. The Microsystems Approach uses universal principles of QI and systems thinking that can readily be applied to other public health issues and is thus a good framework for implementing integrated primary care services.

## Abbreviations

RI: routine immunization
WHO: World Health Organization
HU: health unit
DPT3: third dose of diphtheria, tetanus, pertussis vaccine
DPT1: First dose of diphtheria, tetanus, pertussis vaccine
ARISE-SI: Africa routine immunization systems essentials – system innovation research study
JSI: JSI Research & Training Institute, Inc.
MUSPH: Makerere University School of Public Health
UNEPI: Uganda National Expanded Program on Immunization
MoH: Ministry of health
RED: WHO reaching every district strategy
QI: quality improvement
VHT: village health team member
HMIS: Health Management Information Systems
PDSA: plan-do-study-act
NVIVO: qualitative data analysis software
USAID: United States Agency for International Development
